# Landscape Analysis of Runoff and Sedimentation Based on Land Use/Cover Change in Two Typical Watersheds on the Loess Plateau, China

**DOI:** 10.3390/life12111688

**Published:** 2022-10-24

**Authors:** Xiaojun Liu, Yi Zhang

**Affiliations:** 1 School of Agriculture, Ningxia University, Yinchuan 750021, China; 2 Breeding Base for State Key Laboratory of Land Degradation and Ecological Restoration in Northwest China, Ningxia University, Yinchuan 750021, China; 3 Key Laboratory for Restoration and Reconstruction of Degraded Ecosystem in Northwest China of Ministry of Education, Ningxia University, Yinchuan 750021, China

**Keywords:** land use/cover change, landscape, runoff, sedimentation, Loess Plateau

## Abstract

Understanding sedimentation and runoff variations caused by land use change have emerged as important research areas, due to the ecological functions of landscape patterns. The aims of this study were to determine the relationship between landscape metrics (LMs), runoff, and sedimentation and explore the crucial LMs in the watersheds on the Loess Plateau. From 1985 to 2010, grassland was the dominant landscape in the Tuweihe (TU) and Gushanchuan (GU) watersheds. Unused land and cropland experienced the greatest transformations. The landscape in the study area tended to become regular, connected, and aggregated, represented by increasing of the Shannon’s diversity index and the largest patch index, and decreasing landscape division over time. The landscape stability of the TU watershed was higher than that of the GU watershed. Annual runoff and sedimentation gradually decreased and a significant relationship was found between water and soil loss. Due to larger cropland area and lower landscape stability in the GU watershed, the sedimentation of the two watersheds were similar, even though the runoff in the TU watershed was greater. There were stronger effects of LMs on runoff than that on sedimentation yield. The Shannon’s evenness and the patch cohesion index was identified as the key factors of influencing water and soil loss, which had the greatest effects on runoff and sedimentation. Results indicated that regional water and soil loss is sensitive to landscape regulation, which could provide a scientific understanding for the prevention and treatment of soil erosion at landscape level.

## 1. Introduction

Water and soil loss is a serious problem across the globe and can influence both the biological and physical properties of soil, particularly those related to infiltration rates, nutrient storage, overland flow velocity, and overall soil productivity [1,2,3]. Environmental services and ecological equilibrium are threatened when soil loss is greater than soil production [4]. About 75 billion tons of soil is eroded every year from terrestrial ecosystems across the world [5], and approximately half of the land is affected by water and soil loss [6]. Water and soil loss is, therefore, an important research area.

Previous studies have shown that vegetation can control soil erosion and help retain runoff. Many studies [7,8,9,10] have shown that a high vegetation cover can control water erosion. When rainwater falls on soil, the canopy, roots, and litter components of the vegetation can retain water, weaken the impact of splash erosion, and slow down runoff velocity. These processes of runoff and sediment production are affected by the soil structure, land use type, and vegetation growth patterns [11]. The types and changes of vegetation are the critical factors affecting water and soil loss [12]. A decrease in vegetation cover may result in a growth in erosion problems [13]. For example, deforestation and land reclamation on slopes can accelerate runoff and sedimentation [14]. There is a lot of evidence linking forest clearance and continual cultivation resulting in serious soil erosion [15] because cultivation can change soil properties, such as soil aggregates, permeability, nutrient content, etc., which increases the likelihood of soil erosion [16]. The composition and types of land cover are closely related to runoff process characteristics and sediment yield [17,18]. Excessive land development may weaken the protective action of vegetation on water and soil retention, and encourage runoff and soil erosion. Examples of irrational land uses are planting olive orchards in the Alqueva reservoir region [19], leaving land unused, the inappropriate planting of vineyards [20], and land abandonment. Studies of land use/cover change could help us understand the characteristics of runoff and sedimentation variations, improve eco-environmental stability, and promote the sustainable utilization of water and soil resources.

Water and soil loss is strongly related with land use in landscapes [21]. The relationship between runoff, sedimentation, and vegetation have received attention in recent years [22,23,24]. The spatial configuration and composition of plant communities has become a vital and widely applied factor in studies of the geomorphological processes related to erosion [25]. Patch level landscape analyses have indicated that forests, shrubland, and grassland patches lead to better soil properties and have consequently reduced runoff and sediment yield [26,27]. Changes in landscape pattern could have a large impact on erosion [28]. The current landscape distributions or variations can be characterized by landscape metrics (LMs), which were classified into three levels that are patch, class, and landscape level. Natural conditions and human disturbances can be remarkably reflected by landscape, including configuration, composition, and topography [29]. Assessing water and soil loss via key environmental parameters and quantifying the respective influence of LMs can facilitate the development of water and soil quality management strategies [30]. For example, Silva [31] found that LMs are sensitive to changes in the soil surface when erosion occurs. In the upper Du River watershed, LMs were found to account for almost 65% and 74% of the variation in soil erosion and sedimentation yield, respectively. In a previous study, four main contributing LMs were highlighted that were closely related to the variations in the erosion modulus [32]. Shi et al. [33] identified several LMs that were the main indices that influenced watershed soil erosion and sediment yield using partial least-squares regression. A recent study identified the largest patch index of farmland and the landscape index of forest as the key LMs for preferred landscape planning to protect the water quality [34]. Therefore, LMs can be used for both geomorphic evaluations and quantifications of water and soil when they are subject to runoff and sedimentation inputs [4]. Although quantitative research has analyzed the impact of LMs on soil and water loss, it is still not clear whether the impact is more significant for LMs on soil loss or water loss. In addition, the reason that caused the influence differences of the LMs on water and soil loss between different regions is subject of debate.

Severe soil erosion and water loss in the Loess Plateau in China has attracted widespread attention, since it restrained local socio-economic development and seriously threatened environmental security [35]. It is particularly challenging to establish the relationship between LMs, and runoff and sedimentation on the Loess Plateau. The semi-arid landscapes of the Loess Plateau are water-limited due to the high evaporation and relatively low rainfall. Therefore, this area is particularly sensitive to a deterioration in environmental quality. Investigating the quantitative relationships between LMs and water and soil loss is crucial if soil erosion is to be prevented in these seasonally affected environments [36]. This is of particular importance when attempting to predict runoff and sedimentation.

## 2. Materials and Methods

### 2.1. Research Methodology

By field investigation, data collection, and processing, this paper firstly explored the land use and LM changes of time series in two typical comparative watersheds; secondly, a relation between runoff and sedimentation and LMs at the landscape level was derived by combining ecologically significant LMs; and, finally, the dominant LMs that influence water and soil loss were verified, and the difference exhibited from the two watersheds was discussed.

### 2.2. Study Area

**Figure 1:**
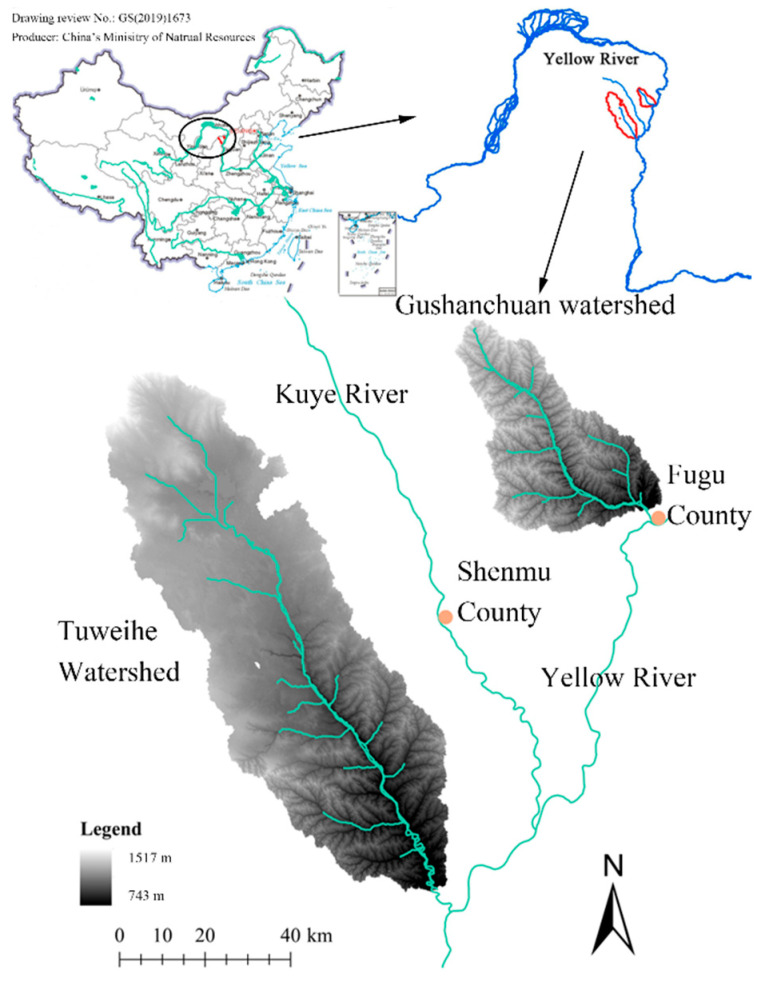
The location of Tuweihe and Gushanchuan watershed, China.

The Tuweihe and Gushanchuan rivers are tributaries of the Yellow River and are located on the right bank of the middle stream. The two watersheds are located between 109°26′–110°05′ E and 38°18′–39°26′ N, and have areas of 4503.40 and 1263.11 km^2^, respectively (Figure 1). Their elevations range from 743 to 1517 m, with a high terrain in the northwest and a low topography in the southeast. They are affected by the northern temperate continental monsoon climate, and the two watersheds are arid and semi-arid regions, with annual mean temperatures of 8.5 and 7.3 °C, respectively. Their annual rainfall amounts are 417.4 and 430 mm, respectively. Concentrated rainfall occurs in the summer, and high evaporation and high intensity storms are the main reasons for the runoff and sedimentation losses in the watersheds. Quaternary loss is widespread in the hilly and gully regions where there is serious wind–water erosion. Two deep fully developed gullies have been cut and their erosion moduli are 2244 and 3299 t km^–2^ a^−^^1^, respectively [37,38].

### 2.3. Research Methods

#### 2.3.1. Data Sources

A digital elevation model (DEM) dataset was provided by the Geospatial Data Cloud, the Computer Network Information Center, Chinese Academy of Sciences (http://www.gscloud.cn, accessed on 28 February 2020). It was processed by the Advanced Spaceborne Thermal Emission and Reflection Radiometer Global Digital Elevation Model (ASTER GDEM) Version 1 and the spatial resolution was 30 m when the Universal Transverse Mercator (UTM, 49N) projection was used. The DEM data were subjected to a mosaic and clipping process, which allowed the study area to be generated. Then, the data were subjected to a depression detention, which meant that the flow generation and drainage network extraction values could be derived and the control watersheds could be created. The hydrological sites at the outlets provided the annual runoff and sedimentation data for 1985–2010.

The land use dataset was provided by the Cold and Arid Regions Science Data Center at Lanzhou, China (http://westdc.westgis.ac.cn, accessed on 8 May 2020). It was funded by major grants from the Chinese Academy of Sciences under the ‘National Resources and Environment Survey and Dynamic Monitoring Using Remote Sensing’ program (96-B02-01). Researchers experienced in interpreting the spectra, texture, and tone of such images created visual interpretations, which were based on Landsat Multispectral Scanner (MSS), Thematic Mapper (TM), and Enhanced Thematic Mapper (ETM) information. Their results were evaluated by field studies and the precision was as high as 95%.

Due to the large study area, the land use type subcategories were merged into the main categories, which provided a scientific rationality and flexibility for landscape change analysis. Six landscape types were established in the geographic information system (GIS) database: cropland, forest land, grassland, water area, urban and rural land, and unused land. The spatial analyst module in the ArcGIS system and conversion tools were used to transform the vector data for land use into raster data for the following analysis.

#### 2.3.2. Research Methods

Describing the characteristics and variations of the landscape using LMs and identifying relationships between landscape patterns and processes are the most common quantitative methods applied in landscape ecology research [39,40]. The Fragstats 3.3 landscape analysis software was used to determine the relevant LMs according to the Fragstats 3.3 operation manual. The LMs were then used to study the pattern properties of the watershed. Fragstats 3.3 can calculate more than 50 LMs. These metrics were divided into three levels representing three different scales. (1) Patch level: this reflected the structural characteristics of single patches in the landscape and provided the computational basis for the other levels. (2) Class level: this reflected the structural characteristics of multiple patches in the landscape. (3) Landscape level: this reflected all the structural characteristics of the landscape. This study used the landscape-level metrics, number of patches, patch density, the largest patch index, the landscape shape index, the perimeter area fractal dimension, the contagion index, the patch cohesion index, the landscape division index, Shannon’s diversity index, and Shannon’s evenness index. These indexes were used because they reflect area, density, proximity, diversity, and divergence [30]. The computing method and ecological significance of each metric are listed in Table 1.

The significance and correlation analyses were undertaken by one-way ANOVA and multiple linear regression using IBM SPSS Statistics Version 2.0 software.

## 3. Results

### 3.1. Land Use Changes in the Watersheds

**Figure 2:**
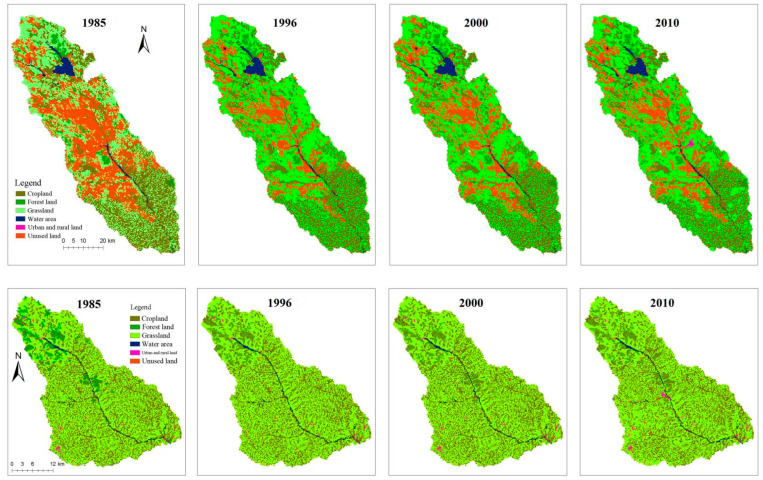
Land use distribution and variations with time in Tu (**up**) and Gu (**down**) watershed.

Table 2 describes the change characteristics of the six land use types over the 25-year period. The area of the Tuweihe River watershed is 4503.40 km^2^ and grassland represented the greatest proportion of the land cover (between 38.13 and 53.49% over the 25-year period), followed by unused land (between 23.08 and 37.90% over the 25-year period). An analysis of the land-use transfer matrix showed that the unused land variance was largest over the study period at 33.82%. Between 1985 and 2010, 35.12% of unused land was turned into grassland, with 67.28% of the conversion occurring between 1985 and 1996 (Figure 2). Furthermore, 92.93 km^2^ of cropland was returned to forest and grassland, with the largest changes occurring between 2000 and 2010 (76.52 km^2^). The proportion of land converted from forest to other land uses was lowest at 5.46%.

The GU watershed has an area of 1263.11 km^2^. The largest proportion of the wetland was grassland (between 61.00 and 62.61% over the 25-year period), followed by cropland (between 30.37 and 32.50% over the 25-year period). From 1985 to 2010, the cropland and unused land areas gradually decreased, and forest land, grassland, and urban and rural land areas increased. The water area was stable but, in the TU watershed, the water area slowly decreased. The cropland area changed the most (52.85 km^2^). Between 2000 and 2010, 49.76 km^2^ of cropland was converted into forest and grassland, while unused land had the highest transfer ratio (51.95%) of all the landscape types.

### 3.2. Land Metrics and Landscape Stability (LS)

Table 3 lists the LMs for the TU and GU watersheds at four time periods. As time progressed, the TU watershed’s number of patches, contagion, and patch cohesion values gradually decreased, whereas the largest patch index, the landscape shape index, perimeter area fractal dimension, landscape division, and Shannon’s diversity values tended to increase. The patch density values remained almost the same over the 25 years. The LMs in the GU watershed changed over the 25-year period but there was no obvious pattern to the variance. The patch density, the largest patch index, perimeter area fractal dimension, contagion, and patch cohesion were all lower in the TU watershed than in the GU watershed.

The cropland, forest, and WAR land use types had the highest LS values. The grassland had the lowest average LS value, particularly between 2000 and 2010. During this period, the character stability (CS) and density stability (DS) of urban and rural land in the TU watershed was 0.409 and 0.881, respectively, followed by a DS of 0.591 for unused land between 1985 and 1996. In the GU watershed, the lowest LS value occurred for forest land, followed by unused land and urban and rural land, which had the lowest values between 1985 and 1996. In general, the LS values for the TU watershed were higher than those for the GU watershed. The LS values for grassland and unused land increased over time, whereas the cropland and urban and rural land declined.

### 3.3. Variation in and the Relationship between Annual Runoff and Sedimentation

**Figure 3:**
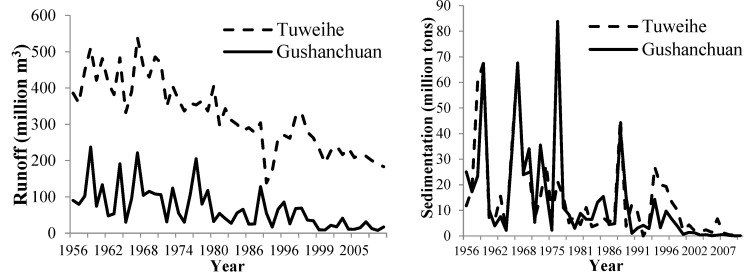
Interannual variation in annual runoff and sedimentation from 1956 to 2010.

The Mann–Kendall trend results showed that runoff and sedimentation tended to decrease over time (*p* < 0.01). The peaks of annual runoff and sedimentation were correlated with each other (Figure 3). Up to 2010, the runoff rate in the TU and GU watersheds was 52.52% and 80.95% of the annual runoff in 1956, respectively. The annual reduction in runoff volume was 3.75 and 1.35 million m^3^, respectively. Sedimentation in 2010 decreased by 97.26% and 99.77%, respectively, relative to the value in 1985, and the average sedimentation reduction per year was 0.21 and 0.46 million tons in the TU and GU watersheds, respectively. The runoff in the TU watershed, which has a larger area than the GU watershed, was higher than in the GU watershed, but annual sedimentation was much the same (1.40 million tons). The rank-sum test showed that there was a break point for annual runoff and the sedimentation process in the two watersheds. In the TU watershed, the break points for runoff and sedimentation occurred in 1981 and 2001, respectively, while it was 1999 for both runoff and sedimentation in the GU watershed.

**Figure 4:**
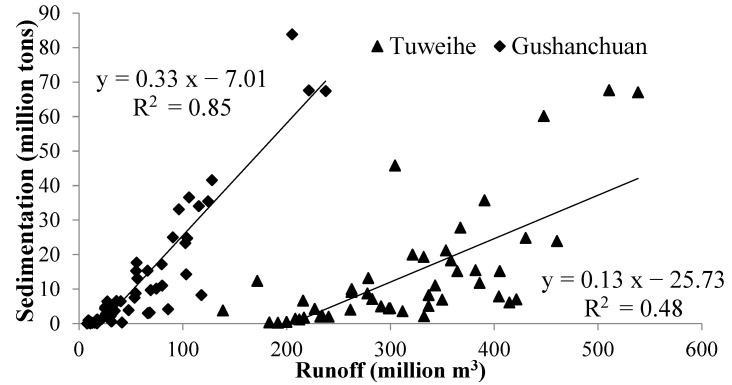
Linear relationship between annual runoff and sedimentation.

The Pearson’s correlation analysis revealed that there was a strong positive relationship between runoff and sedimentation in the two watersheds (*p* < 0.01, Figure 4). The coefficient of determination for the TU watershed, which has a higher landscape diversity, was 0.48, whereas the coefficient of determination for the GU watershed was 0.85. The sediment-carrying capacity of the runoff (i.e., the slope of the regression line) in the GU watershed was greater than in the TU watershed carrying capacity. This showed that the sedimentation yields of the GU watershed were similar to those of the TU watershed, even though there was substantially less runoff (19.27% of that in the TU watershed).

### 3.4. Response Relationships between Runoff, Sedimentation, and LMs

A Pearson’s analysis was conducted to determine the effects of landscape on runoff and sedimentation (Table 4). The results showed that there were significant correlations between the factors. More LMs were significantly (*p < 0.05*) or highly significantly (*p < 0.01*) correlated with annual runoff. When patch density, contagion, and patch cohesion rose, the annual runoff declined. In contrast, the LMs related to landscape diversity, such as Shannon’s diversity and Shannon’s evenness, were positively associated with annual runoff and sedimentation (*p < 0.01*). These relationships implied that the increase in patch density and area led to a decrease in runoff. Furthermore, contagion and patch cohesion had direct impacts (*p < 0.05*) on erosion (coefficients of determination of 0.773 and 0.738, respectively).

## 4. Discussion

The Chinese government initiated the Grain for Green Program (GGP) in 1999 and this nationwide project has gradually changed the national land use structure [41]. The Loess Plateau was particularly affected by the program because it was considered as a priority region [42]. Large areas (Table 2) have been converted to various alternative landscapes in the study watersheds. More check dams were constructed in Tu watershed than in Gu watershed, which play a vital role in intercepting sediment. It was confirmed by the lower coefficient determination in the relationship between runoff and sedimentation in TU watershed (Figure 3). Both watersheds have been subjected to continuous deforestation and conversion of cropland to forest. In the process, patch connectivity developed, which led to species migration and other ecological processes. This was confirmed by the increase in the largest patch index, patch cohesion, and contagion values. The landscape shape index decrease in the TU watershed showed that many patches were affected by anthropogenic activities, which led to a regular and simple patch pattern. This was confirmed by the decrease in the perimeter area fractal dimension values.

In the TU watershed, number of patches decreased with time, but patch cohesion and contagion increased, which indicated that good connectivity was formed by merging a landscape type with species migration and other ecological processes [43]. The variable decreases in landscape shape index and perimeter area fractal dimension illustrated that many patches were being affected by human activities, which also showed that the landscape consisted of regular and simple patches. Large stretches of grassland were recreated in 1996, which led to the lowest value for Shannon’s diversity. The lower patch density and area parameters resulted in a complex landscape system in the TU watershed. Therefore, the Shannon’s diversity value for the TU watershed was higher than that of the GU watershed. The LS for the urban and rural land in the TU watershed decreased over time due to increased anthropogenic activities (Table 5). More than five programs, including the conversion of cropland to forests program, have been initiated since 1978 in an attempt to control desertification and soil loss. Furthermore, afforestation has also increased in China over the last decade [44]. Therefore, the LS values for grassland and unused land increased after 2000 and interference due to human activities declined across the two landscape types.

The cropland landscape was the key factor affecting soil conservation [45] in the study area and there were more check dams in the TU watershed according to the field investigation, which caused the annual sedimentation yield in the TU watershed to be similar to the yield for the GU watershed, even though the annual runoff in the TU watershed was significantly higher (*p* < 0.01, Figure 3). Fragmented natural landscape indicated intensive agricultural activities, which caused more serious erosion and soil nutrient loss [46]. Higher landscape stability of TU watershed further confirmed its controlling function on sedimentation with higher runoff. In addition, the variation coefficient for annual sedimentation was higher than that for annual runoff, which indicated that the sedimentation was more susceptible to environmental effects than runoff.

Runoff and the sediment deposited in water is contained by the spatial pattern of the landscape [47]. The LMs synthesize the retardation capacity and spatial position, and reflect the potential risk of water and soil loss. For example, the Shannon’s diversity value is not a biodiversity metric but, rather, focuses on the unbalanced distribution of various patch types in the landscape. In the study area, the diversity of land uses and low degree of landscape fragmentation exerted significant positive influences on runoff (*p* < 0.01, Table 4). The contagion and patch cohesion values had significant negative correlations with annual runoff and sedimentation (*p* < 0.05), which indicated that water and soil loss decreased when external and internal patch connectivity improved. Most LMs were significantly related to annual runoff, which showed that the landscape had a greater effect on runoff than sedimentation. This means that it can be used as an ecological indicator to predict runoff, relative to sedimentation. The LS changes showed that there was an abrupt runoff change in 1981, which the land use data did not show. The sedimentation-to-runoff ratio was lowest in 2001, and the LS values for forest land and grassland were also at their lowest compared to 1985–1996 and 2000–2010 (Table 5). In the GU watershed, the lowest LS for forest land occurred in 1999 (1996–2000), which indicated that there had been a sharp increase in forest land. This could have caused the sharp decrease in runoff and sediment deposition in 1999. It, therefore, appears that a breakpoint usually occurs when the LS for forest land and grassland is small, which suggests that there has been a major expansion in these types of land use.

A Pearson’s analysis was conducted to determine the factors that most strongly influence the annual decrease in runoff (DR) and sedimentation (DSe) (Table 6). The results showed that the correlations between DSe and LS, and the different land use types were not significant (*p* > 0.05). However, the DR was positively correlated to the DS for grassland (P = 0.740, *p* < 0.05), which meant that annual runoff in the watershed could be significantly reduced if the grassland had a high DSe. When the grass patches were highly connected, runoff could be effectively intercepted [48]. Therefore, the LMs and LS effects on runoff became more significant.

A stepwise regression analysis was used to determine the most influential variables that were not strongly correlated with one another [49]. Every independent variable was subjected to an F test and then deleted if the F-value showed that the variable was not significant. Furthermore, the previous variable was deleted if the F-value was not significant when a new independent variable was added to the set. This algorithm was repeated until no independent variable could be added or deleted. The optimal regression model was then established after applying this method (Table 7). The variance inflation factors (VIF) were 0.446 and 2.244 for the TU and GU watersheds, respectively, which meant that the collinearity hypothesis could be rejected. The significance values were all lower than 0.05. Therefore, the selected LMs (Shannon’s evenness and patch cohesion) were the most significant factors affecting annual runoff and sedimentation. When the dominant landscapes had greater ecological benefits, the annual runoff decreased. Furthermore, measures that promote the value of patch cohesion should be taken if the interception function of water and soil loss is to be improved.

This study applied DEM dataset processed by ASTER GDEM for landscape analysis to assess water and soil loss. Although it has been proved to be an appropriate application in the field of erosion estimation [50] and provided scientific basis for soil erosion prevention and land use management, it is still a worthy study to investigate the result difference with finer or coarser resolution. In addition, the relationship between LMs and erosion we established and discussed was based on the dataset collected in the focalized regions, which are typical watershed on the Loess Plateau. Considering the extension of the scientific research, more analysis should be executed with larger scale and different regions. There is, of course, conventional existing research that concluded that LMs, e.g., Shannon’s evenness and patch cohesion, were significantly correlated with soil erosion in the whole region of Loess Plateau [51]. In terms of driving factors, vegetation cover and landscape variables are not the only factors that influence the erosion process; soil properties, climatic conditions, etc., also play a vital role in water and soil loss [52]. Therefore, to increase the validity of analysis and deepen the understanding of soil erosion processes, more related variables should be considered in further related research.

## 5. Conclusions

From 1985 to 2010, the landscape of the study area tended to become regular, connected, and aggregated, while the annual runoff and sedimentation values gradually decreased. The LS values for grassland and unused land gradually increased, but they decreased for cropland and urban and rural land due to human activities. Due to larger cropland area and lower landscape stability in the GU watershed than that in the TU watershed, the annual sedimentation for the two watersheds was similar, even though the annual runoff in the TU watershed was greater than that in the GU watershed. The annual runoff was significantly and positively correlated with sedimentation (*p* < 0.01), and the coefficient of determination for the TU watershed (0.48) was substantially lower than that for the GU watershed (0.85). The LMs had more significant influences on runoff than on sedimentation (*p* < 0.01), especially given that density stability for grassland could significantly decrease the runoff in the study area. The Shannon’s evenness and patch cohesion were the crucial factors for affecting water and soil loss, and the measures involving landscape and land use could have a greater influence on runoff than on sedimentation.

## Figures and Tables

**Table 1:** Description and ecological significance of landscape metrics in this study.

Landscape Index	Formula	Physical Significance	Ecological Significance
Number of patches	N	N: numbers of the patches; Unit: a	the whole numbers of the patches in the landscape
Patch density	$\frac{1}{A}\overset{M}{\underset{j=1}{\sum }}N_{i}$	M: types of landscape in the study area; A: total area of the landscape; N: ditto; Unit: a/km^2^	degree of fragmentation and spatial heterogeneity; reflecting the human disturbance to a certain extent
Largest patch index	$\frac{Max\left(a_{1},a_{2},\ldots .a_{n}\right)}{A}\left(100\right)$	a_i_: area of the “i” patch; A: ditto; Unit:%	help to confirm the dominant type; the variation could change the intensity and frequency of the disturbance that reflect the direction and strength of human activities
Landscape shape	$\frac{0.25E}{\sqrt{A}}$	E: perimeter of patches; A: ditto	the bigger the value, the more complicated the patches
Perimeter area fractal dimension	2{ni∑i=1m∑j=1n(lnPij×lnAij)−(∑i=1m∑j=1nlnPij)(∑i=1m∑j=1nlnAij)}(ni∑i=1m∑j=1nlnP2ij)−(∑i=1m∑j=1nlnPij)	P_i_: proportion of i type in the whole landscape; g_ik_: number of patches between i and k type; m: ditto	the bigger the value, the greater the fragmentation of patches
Contagion	$\left[1+\frac{\sum_{i=1}^{m}\sum_{j=1}^{n}\left(P_{i}\right)\frac{g_{ik}}{\sum_{k=1}^{m}g_{ik}}\times ln\left(p_{i}\right)\frac{g_{ik}}{\sum_{k=1}^{m}g_{ik}}}{2ln\left(m\right)}\right]\times 100$	M: ditto; Pij: probability of the random selected two adjacent grid belonging to i and j type; Unit: %	the degree of agglomeration and extending tendency of different patch type
Patch cohesion	$\left(1-\frac{\sum_{J=1}^{m}P_{\mathrm{ij}}}{\sum_{j=1}^{m}P_{\mathrm{ij}}\sqrt{a_{\mathrm{ij}}}}\right){\left(1-\frac{1}{\sqrt{A}}\right)}^{-1}g\left(100\right)$	P_ij_: perimeter of patch ij; a_ij_: ditto; A: ditto; Unit: %	spatial connection between a type of patch with the adjacent patches
Landscape division	$\left[1-\overset{n}{\underset{j=1}{\sum }}\left(\frac{a_{ij}}{A}\right)\right]$	a_ij_: area of patch j with landscape i; A: ditto	fragmentation of the landscape
Shannon’s diversity	$-\overset{m}{\underset{i=1}{\sum }}\left[P_{i}\ln\left(P_{i}\right)\right]$	P_i_: proportion of i type in the whole landscape; i: numbers of patch	complicity and heterogeneity of the landscape, emphasizing the contribution of rare patch to the information
Shannon’s evenness	$\frac{-\sum_{i=1}^{m}\left(P_{i}\ln P_{i}\right)}{\ln m}$	ditto	reflecting one or several dominant landscapes

**Table 2:** The change variations of land use with time in the study area (km^2^).

**Land Use**	**Tuweihe Watershed**				**Gushanchuan Watershed**			
	1985	1996	2000	2010	1985	1996	2000	2010
**Cropland**	1129.26	1134.52	1116.35	1086.42	410.49	405.94	409.21	383.59
**Forest land**	203.77	201.87	204.74	212.33	60.47	48.41	64.45	72.91
**Grassland**	1681.02	2251.95	2124.38	2175.39	772.61	790.87	770.47	785.15
**Water area**	106.10	105.44	104.98	102.97	12.37	12.82	12.10	12.22
**Urban and Rural land**	8.70	8.62	9.03	18.65	6.12	4.51	6.32	8.53
**Unused land**	1374.55	801.00	943.91	909.61	1.05	0.56	0.56	0.55

**Table 3:** Annual variations in landscape indices (units: see Table 1).

Watershed	Time	Number of Patches	Patch Density	Largest Patch	Landscape Shape	Perimeter Area Fractal Dimension	Contagion	Patch Cohesion	Division	Shannon’s Diversity	Shannon’s Eveness
**Tuweihe**	1985	1393	0.31	20.30	36.48	1.60	36.47	97.79	0.91	1.32	0.734
	1996	1332	0.30	41.04	35.32	1.58	39.82	98.72	0.80	1.24	0.690
	2000	1343	0.30	37.39	36.16	1.58	38.46	98.60	0.83	1.27	0.706
	2010	1340	0.30	34.03	35.94	1.57	38.36	98.44	0.86	1.27	0.707
**Gushanchuan**	1985	938	0.74	61.00	37.14	1.68	53.34	99.18	0.62	0.89	0.495
	1996	909	0.72	62.50	36.34	1.69	55.22	99.21	0.61	0.85	0.476
	2000	959	0.76	60.81	37.27	1.69	53.07	99.17	0.63	0.89	0.498
	2010	928	0.74	61.79	35.73	1.68	52.59	99.14	0.62	0.91	0.506

**Table 4:** Regression relationships between LMs, runoff, and sedimentation.

	LMs	Regression Equation	R^2^	Sig.
**Runoff**	Patch density	−4.457PD + 5.010	0.916	0.003 **
	Contagion	−0.113contagion + 8.191	0.738	0.028 *
	Patch cohesion	−0.717cohesion + 71.936	0.773	0.021 *
	Shannon’s diversity	3.312SHDI−3.361	0.930	0.002 **
	Shannon’s evenness	12.280SHEI−4.937	0.934	0.002 **
**Sedimentation**	Contagion	−0.006contagion + 0.474	0.693	0.04 *
	Patch cohesion	−0.043cohesion + 4.294	0.760	0.024 *

* Significant at *p* < 0.05; ** significant at *p* < 0.01.

**Table 5:** Landscape stability variation characteristics.

**Landscape**	**Year**	**Tuweihe Watershed**		**Gushanchuan Watershed**	
		CS	DS	CS	DS
**Cropland**	1985–1996	0.996	0.995	0.981	0.973
	1996–2000	0.982	0.978	0.989	0.987
	2000–2010	0.952	0.932	0.909	0.881
	1985–2010	0.938	0.914	0.914	0.893
**Forest land**	1985–1996	0.973	0.954	0.621	0.441
	1996–2000	0.965	0.943	−0.089	−0.845
	2000–2010	0.959	0.956	0.705	0.541
	1985–2010	0.952	0.948	0.482	0.169
**Grassland**	1985–1996	0.687	0.714	0.885	0.791
	1996–2000	0.956	0.967	0.813	0.648
	2000–2010	0.936	0.896	0.910	0.841
	1985–2010	0.683	0.661	0.871	0.761
**Water area**	1985–1996	0.951	0.912	0.962	0.959
	1996–2000	0.984	0.969	0.961	0.978
	2000–2010	0.969	0.955	0.963	0.934
	1985–2010	0.932	0.898	0.994	1.000
**Urban and rural land**	1985–1996	0.995	1.000	0.835	0.934
	1996–2000	0.968	0.985	0.738	0.880
	2000–2010	0.409	0.881	0.652	0.651
	1985–2010	0.362	0.865	0.598	0.589
**Unused land**	1985–1996	0.591	0.598	0.519	0.506
	1996–2000	0.883	0.945	0.999	1.000
	2000–2010	0.977	0.990	0.892	0.800
	1985–2010	0.676	0.689	0.564	0.608

**Table 6:** Pearson’s analysis between the DR, DSe, and LS values for the different land use types.

		Cropland CS	Cropland DS	Forest Land CS	Forest Land DS	Grassland CS	Grassland DS	Water Area CS	Water Area DS	URL CS	URL DS	Unused Land CS	Unused Land DS
**DR**	Pearson Correlation	0.001	0.016	0.275	0.268	0.586	**0.740** *	0.363	0.145	0.338	0.127	0.347	0.375
	Sig. (2-tailed)	0.998	0.970	0.510	0.520	0.127	**0.036**	0.377	0.732	0.412	0.765	0.400	0.359
**DSe**	Pearson Correlation	−0.329	−0.369	−0.154	−0.139	−0.180	−0.378	−0.319	−0.162	−0.636	−0.336	0.013	−0.098
	Sig. (2-tailed)	0.427	0.368	0.716	0.742	0.670	0.356	0.442	0.702	0.090	0.417	0.976	0.818

* Correlation is significant at the 0.05 level (two-tailed). DR and DSe mean decrease in annual runoff and sedimentation, CS and DS mean stability of character and density. URL means urban and rural land.

**Table 7:** Optimal regression model for LMs, runoff, and sedimentation.

Dependent	Model		Unstandardized Coefficients		Standardized Coefficients	t	Sig.	Collinearity Statistics	
			B	Std. Error	Beta			Tolerance	VIF
**runoff**	1	(Constant)	−4.937	0.876		−5.636	0.005		
		Shannon’s evenness	12.280	1.630	0.967	7.534	0.002	1.000	1.000
	2	(Constant)	25.492	6.921		3.683	0.035		
		Shannon’s evenness	8.895	1.032	0.700	8.618	0.003	0.446	2.244
		Patch cohesion	−0.292	0.066	−0.358	−4.403	0.022	0.446	2.244
**sedimentation**	1	(Constant)	4.294	1.184		3.627	0.022		
		Patch cohesion	−0.043	0.012	−0.871	−3.554	0.024	1.000	1.000

## Data Availability

Not applicable.
